# Supplementary material for: Are carbon emissions trading and green financial instruments synergistic? -Comprehensive quantitative research based on content analysis
Source: PLoS One. 2024 Mar 7;19(3):e0298601. doi: 10.1371/journal.pone.0298601 (PMC10919601; doi:10.1371/journal.pone.0298601)
Supplement: S1 Data — (ZIP) [file pone.0298601.s001.zip › Data/地级市Z_F工作报告.zip!/║■▒▒/╧σ╤⌠╩╨/└┤╫╘╣1⁄2╓┌║┼í╛┬φ┐╦╩2╛▌═°í┐_2012.docx]

各位代表：

　　现在我代表市人民政府向大会作工作报告，请予审议，并请政协委员和其他列席人员提出意见。

　　一、关于过去四年的工作

　　过去四年，是襄阳改革开放以来经济社会发展最快、人民群众受益最多的时期之一。四年来，在市委的坚强领导下，全市上下深入贯彻落实科学发展观，有效应对国际金融危机冲击，努力化解自然灾害等不利影响，奋力推进省域副中心城市建设，全市经济社会发展实现了历史性跨越。

　　——经济发展迈入快车道，综合实力显著增强。固定资产投资两年翻番、规模以上工业总产值三年翻番、县域经济三年翻番目标全面实现。2011年全市实现生产总值2132.2亿元，是2007年的2.7倍，占全省的比重由8.4%提高到10%以上；规模以上工业产值3276.5亿元，是2007年的4.1倍；四年累计完成固定资产投资2919亿元，是前四年的3.9倍；完成地方一般预算收入97.4亿元，是2007年的3.3倍。经济结构明显优化。三次产业比例由2007年的17.8∶43.6∶38.6调整为13.7：57.1：29.2。工业主导地位进一步突出。汽车及零部件和农产品加工业成为千亿级产业，新能源新材料、装备制造、电子信息、医药化工等产业呈现良好发展势头，规模以上工业增加值占生产总值的比重达到48.5%，比2007年提高15.5个百分点，我市成为国家节能与新能源汽车示范推广试点城市、国家新型工业化(新能源汽车)示范基地和军民结合产业示范基地。农业大市地位显著提升。综合生产能力不断增强，粮食生产实现“八连增”，农产品加工业产值和农业增加值均居全省第二位。服务业发展加快。商贸、物流市场主体快速成长，文化旅游业突破性发展，社会消费品零售总额增幅连续四年高于全省平均水平。县域经济实力快速提升，在全省考核中连续三年大幅进位，襄州区和枣阳市跻身全省二十强。

　　——发展方式转变加快，可持续发展能力明显提升。科技创新能力不断增强。认定省级以上重大科技成果525项，获得国家和省科学技术奖40项、授权专利5303件；高新技术和战略性新兴产业蓬勃发展，新能源汽车关键零部件产业成为国家创新型产业集群，高新技术产业增加值居全省第二位；高新区成为全国首批两个国家级创新型特色园区之一，我市被评为“全国科技进步示范市”和“全省科技创新先进市”。人才强市战略积极推进。“隆中人才支持计划”深入实施，引进国家“千人计划”和省“百人计划”高端人才5人、国家级专家18人、硕士以上人才479人。“两型社会”建设扎实推进。预计省政府下达的节能减排目标全面实现。森林覆盖率达到42.6%，市区空气质量优良天数保持在310天以上，汉江襄阳段水质稳定在二类水平。我市成为国家可再生能源建筑应用示范城市、再生资源市场体系建设试点城市和节水型社会建设示范市，谷城再生资源园区成为中部六省唯一的国家级“城市矿产”示范基地。

　　——招商引资实现历史性突破，开放水平明显提高。全市招商引资累计到位资金突破1000亿元。2011年新注册招商项目总投资和央企签约项目总投资均突破1000亿元。利用外资大幅增长，累计批准设立外商投资企业124家，实际外商直接投资11.5亿美元，分别是前四年的1.4倍和3.65倍。开放平台明显优化，全省唯一的二类铁路口岸、出口配送型监管仓库和鄂西北唯一的物流保税仓库相继建成，2011年完成外贸出口总额7.7亿美元，是2007年的2.8倍。

　　——各项改革深入推进，体制机制创新取得积极成效。“阳光新政”深入实施，新一轮市、县政府机构改革全部到位，行政审批制度改革不断深化，“治庸问责”工作深入开展，行政服务环境明显优化。文化、医药卫生和食品药品监管体制改革走在全省前列。国企改革和集体林权制度主体改革基本完成，农村土地二轮延包依法规范，土地流转有序推进，非公有制经济发展明显加快。金融创新取得突破，多层次金融市场体系初步形成，银行业金融机构由7家发展到18家，2011年金融机构净增贷款141.7亿元，是2007年的3.9倍；股权融资和债券融资取得积极成效，回天胶业、台基股份、骆驼股份成功上市，全市上市公司达到8家，成功发行两期城投债25亿元，均居全省第二位。土地利用管理创新成效明显，保障了重大项目建设用地需求。

　　——城镇化进程不断加快，城市功能日益增强。城镇化率由2007年的44.9%提高到50.9%。市、县两级累计投入300多亿元，大力推进城市基础设施建设；主城区建成区面积扩大20平方公里，城市功能不断增强；新建、扩建15个开发区(工业园区)100平方公里，高新区成为千亿级开发区，经开区晋升为国家级开发区，鱼梁洲成为长江流域首家获准开发利用的洲岛型开发区；改革城管体制，实施“城管革命”，强力查处违法建设，狠抓拆墙透绿等“八项集中整治”，城市面貌明显改观，顺利通过湖北省卫生城市复查验收，我市被评为全国无障碍建设示范城市。武汉至安康铁路复线襄阳段建成通车，襄北编组站和襄阳东站建设顺利推进，崔家营航电枢纽投入运营，谷(城)竹(山)、麻(城)竹(溪)高速公路襄阳段开工建设，全市国、省干线公路全部达到二级以上标准，区域性交通枢纽地位明显提升。农村水利、电力、交通等基础设施全面升级，所有行政村基本通达水泥路，10.5万贫困人口的温饱问题和162.4万人的饮水安全问题得以解决，25.2万个农户用上沼气，37个村成为省级宜居村庄，9个乡镇成为全省新农村建设试点，南水北调移民安置任务全面完成，新农村建设“三年明显变化”目标全面实现。

　　——社会建设持续加强，人民生活水平明显改善。坚持每年集中财力办好“十件实事”，解决了一大批人民群众关注的热点难点问题。就业和社会保障水平显著提升。城镇登记失业率控制在4.2%以内，城镇各项社会保险覆盖面持续扩大，5个县(市)区成为国家城乡居民养老保险试点，城镇居民医疗保险、新型农村合作医疗实现愿保尽保，城乡低保对象实现应保尽保，城镇离退休职工、低保对象、优抚对象待遇和城镇职工最低工资标准大幅提高，27万城镇低收入居民住房困难得到解决。城乡居民收入较快增长。2011年城镇居民人均可支配收入和农民人均纯收入分别达到15352元和7549元，年均增长12.5%和16.4%。公共服务水平不断提高。资助贫困学生基本实现全覆盖；学前教育不断加强，70%的乡镇均办起公立幼儿园，义务教育均衡发展整体推进，免费义务教育全面实施，高中阶段教育基本普及，基础教育质量居全省前列，职业教育发展加快，高等教育规模不断扩大，市域高校达到5所。卫生工作走在全省前列，主要健康指标优于全省、全国平均水平，援非医疗队得到国家卫生部表彰。全民健身活动蓬勃开展。省政府下达的计划生育责任目标全面完成。办理困难群众法律援助案件5800多件，妇女儿童、老年人、残疾人和农民工合法权益得到维护。加强和创新社会管理取得突破，信访维稳工作得到中央领导充分肯定，创新社会管理经验被中央综治办专题推广，安全生产、消防安全、食品药品安全监管有力，应急管理水平明显提升，平安创建工作富有成效，人民群众安全感普遍增强，被评为“全国社会治安综合治理优秀市”和“全省安全生产责任目标考核优秀市”。

　　——民主法制建设不断加强，精神文明建设取得明显成效。自觉接受市人大和市政协的监督，认真办理人大代表议案、建议和政协建议案、提案；规范决策程序，坚持问计于民，促进了科学决策。电子政务建设初见成效。政务公开深入推进，行政监察力度不断加大，廉政建设不断加强。积极推进依法治市，规范行政执法行为，依法行政水平不断提高，被评为“全省法治建设先进城市”。精神文明建设成效显著。公益性文化事业取得长足发展，文化信息资源共享工程、乡镇文化站、农家书屋基本实现全覆盖，广播电视实现村村通，人民群众的精神文化生活不断丰富。我市连续两届荣膺“湖北省文明城市”，被评为“全国创建文明城市工作先进城市”、“全国双拥模范城”和“全国民族团结进步模范市”。

　　刚刚过去的2011年，我市经济社会发展实现了“十二五”良好开局。地区生产总值、规模以上工业增加值、全社会固定资产投资、社会消费品零售总额、地方一般预算收入、外贸出口、实际外商直接投资、城镇居民人均可支配收入和农民人均纯收入同比分别增长16%、26.8%、41.3%、19%、60.8%、38.2%、15%、15.1%和18.6%。

　　与此同时，统计、审计、物价、人防、气象、档案、史志、国防后备力量建设和支援西藏、四川、新疆、恩施等各项工作都取得了突出成绩。

　　各位代表，过去四年取得的成就，得益于省委、省政府和市委的正确领导，得益于市人大、市政协的监督和支持，凝聚着全市人民的智慧和汗水。在此，我代表市人民政府，向全市人民，向所有纳税人，向各位人大代表、政协委员，向各民主党派、工商联、人民团体和无党派人士，向驻襄人民解放军、武警官兵和公安干警，向所有关心支持襄阳发展的同志们、朋友们，表示衷心的感谢并致以崇高的敬意！

　　过去四年的发展成就令人鼓舞，但是我们也清醒地看到，经济社会发展中的矛盾和问题仍然较多。主要是：综合经济实力不强，做大总量与提升质量的任务十分艰巨；经济外向度不高，口岸建设尤其是航空口岸建设滞后；发展方式仍较粗放，龙头产业大而不强，支柱产业支撑力不足，新兴产业刚刚起步，整体竞争力有待提升；农业基础设施依然十分薄弱，提高农业综合生产能力、加快建设现代农业任重道远；现代服务业发展明显滞后，尤其是物流瓶颈制约日益明显；城市规划建设管理水平不高，城市功能还不完善；社会建设欠账较多，公共服务和民生保障水平还需进一步提高；少数政府部门工作人员素质不高、作风不实，庸懒散软的问题还比较突出，发展环境有待进一步改善；等等。对此，我们将采取有效措施，全力加以解决。

　　二、关于今后五年的目标任务

　　未来五年，是加快建设“四个襄阳”，推进省域副中心城市跨越式发展，向现代化区域中心城市迈进的关键时期。当前，我们面临前所未有的挑战。世界经济风险增多，外需下降导致国内市场竞争加剧，我国经济增长下行压力和物价上涨压力并存，要素制约日益趋紧，我市经济社会发展中的矛盾和困难依然较多，要实现跨越式发展的目标任务十分艰巨。对此，我们必须保持清醒头脑，增强忧患意识，积极有效应对。与此同时，我们更要看到前所未有的发展机遇。国家宏观政策紧中趋活，稳中求进，促进中部崛起和振兴老工业基地战略深入实施，将形成长期有利的发展环境。省委、省政府加快实施“一主两副”发展战略，带来了千载难逢的历史机遇。随着两条高速铁路、北煤南运铁路专线、五条高速公路、汉江航运体系建设及襄阳机场改造等一大批重大交通设施项目的陆续实施，我市区域性综合交通枢纽功能将显著提升，对外开放环境将显著改善。世界经济结构调整力度明显加大，尤其是日韩、台港和沿海地区产业转移规模更大、层级更高、步伐更快，为我市引进大企业、培育大产业提供了重大契机。近几年来，一批重大基础设施和产业项目相继建成，一批优势产业和企业正在崛起，为跨越式发展奠定了坚实的基础。市第十二次党代会吹响了向现代化区域中心城市进军的号角，全市上下跨越式发展的氛围进一步浓厚。只要我们坚定信心，抢抓机遇，克难攻坚，一定能够开创襄阳科学发展和跨越式发展新局面。

　　今后五年政府工作的指导思想是：以邓小平理论和“三个代表”重要思想为指导，深入贯彻落实科学发展观，按照市第十二次党代会总体部署和建设“四个襄阳”的总体布局，坚持开放引领，强化创新驱动，壮大产业支撑，推进绿色发展，突出民生优先，促进和谐繁荣，全面提升城市综合竞争力，奋力推进省域副中心城市跨越式发展，加快建设现代化区域中心城市。

　　根据上述指导思想，未来五年的奋斗目标是：

　　——打造区域经济中心。形成区域经济中心的产业规模、辐射功能、结构优势和综合竞争力，经济总量达到全省六分之一左右，规模以上工业产值突破万亿元，综合经济实力进入中部城市“第一方阵”。

　　——打造区域中心城市。大都市城镇体系基本建立，城乡一体化走在区域前列，城镇化率达到56%以上，200平方公里、200万人口规模的现代化特大城市基本建成，城市功能配置形成优势，建设品质大幅提升，特色魅力充分彰显，能量层级全面提高，区域龙头地位凸显，城市综合竞争力进入全国百强。

　　——打造中国文化名城。历史文化资源得到充分保护和利用，襄阳文化特色充分彰显，文化创造力、影响力明显增强。城市精神塑造富有成效，全民文明素质不断提高。文化旅游产业突破性发展，文化和旅游产业增加值占生产总值的比重高于全省平均水平。成为文化事业与文化产业繁荣发展的文化旅游名城。

　　——打造绿色生态家园。人与自然和谐发展，人口与计划生育和节能减排指标控制在省定目标以内，生态环境质量持续改善，森林覆盖率达到45%以上，林木总蓄积量达到2700万立方米以上，汉江干流水质稳定在二类水平，主城区空气质量良好天数保持在310天以上，成为国家级可持续发展试验区；社会事业全面发展，惠及全民、水平更高的基本公共服务体系和社会保障体系全面建立，城镇登记失业率控制在4.5%以内，城镇居民人均可支配收入和农民人均纯收入年均分别增长12.6%和12.8%以上，安全生产指标控制在省定目标以内，保持社会和谐稳定，人民群众幸福感普遍增强。

　　根据上述指导思想和奋斗目标，今后五年政府工作将突出抓好六大战略任务：

　　(一)加快做大总量、提升层次，增强产业核心竞争力

　　加快建设“万亿工业强市”。着力实施主导产业培育工程、骨干企业成长工程和千亿级产业集聚区建设工程。改造提升传统产业，突破性发展高新技术和战略性新兴产业，突出做大做强“一个龙头、六大支柱”产业，力争汽车及零部件产业产值突破4000亿元，农产品加工业产值突破2000亿元，新能源汽车、新能源新材料、装备制造、电子信息、医药化工等产业向千亿级迈进，着力培育20家产值过100亿元、50家过50亿元、200家过10亿元、1000家过亿元的骨干企业。大力发展乘用车、商用车、专用车、新能源汽车及零部件产业，努力建设中国汽车新城；大力发展太阳能光伏、光伏建筑一体化和新能源装备制造业集群，努力建设中国新能源之都；大力发展航空航天装备、智能制造装备、轨道交通装备等高端装备制造业集群，努力建设全国重要的现代装备制造业基地；大力发展汽车电子和光电产业，培育车联网、物联网、智能电网、半导体照明、新型显示、通信电子等一批产值过百亿元的特色产业集群，努力建设全国重要的电子信息产业基地。着力打造千亿级产业集聚区，把高新区建设成为国家创新型特色示范园区和中部领先的科技新城，规模工业产值突破5000亿元；把经开区建设成为优势产业、新兴产业聚集发展的重要平台，使两个开发区尽快步入国家级开发区“第一方阵”。推进各县(市)开发区和各类工业园扩容提质，支持保康发展“飞地经济”，办好余家湖保康工业园，打造一批千亿级开发区、百亿级工业园区。

　　加快建设区域性服务业中心。以创建国家级现代服务业综合改革示范区为抓手，推进服务业转型升级。优化服务业布局，推动高端服务业向汉江沿岸和中央商务区聚集，大型生产性服务业向近郊布局，生活性服务业在区域均衡分布。规划建设金融商务区，打造一批特色商业街区和大型都市商圈。大力发展餐饮购物、医疗保健、休闲娱乐、社区服务等生活性服务业。规范发展房地产业。突破性发展电子商务、云计算、物联网、文化创意等新兴服务业，加快建设中国移动湖北公司襄阳创新基地“四个中心”项目，打造“数字襄阳”、“智慧城市”、“襄阳云谷”。顺应现代服务业与先进制造业融合的趋势，完善现代物流体系，规划建设一批大型物流园区，大力发展保税物流；引进和发展一批研发设计、检验检测、品牌营销等与产业配套的公共服务平台，积极发展高新技术服务业；加快发展金融证券、商务会展和中介服务业。积极创建区域性服务外包基地，建设中国汽车会展中心、中国汽车服务外包基地、区域性金融服务外包基地和区域性总部基地。努力把襄阳建成区域性商贸中心、物流中心、金融中心和信息中心。

　　加快推进农业大市向农业强市跨越。重视和加强“三农”工作，坚持“三化同步”、“两增同步”，加大强农惠农富农政策支持力度。用工业化理念、产业化思路抓农业，加快转变农业发展方式，大力实施百亿斤粮食、千亿级农产品加工、千万头牲畜养殖和千亿元林产业“一百三千”工程，发展农业物流贸易，构建现代农业产业体系。加大龙头企业的扶持和引进力度，培植奥星粮油、襄大农牧、襄阳正大、湖北梅园等一批产值过100亿元的龙头企业，培育粮食、油料、畜禽、纺织、林特等5个产值过500亿元的农产品加工业集群。加快推进农业生产标准化、专业化、规模化，抓好农业科技创新和技术推广，发展农业机械化，提高农业现代化水平。多渠道增加农业投入，加强农田水利基本建设，完善农业防灾减灾体系，增强农业综合生产能力。把襄阳建成全国现代农业示范区、现代水利示范区、优质农产品生产加工基地和区域性农产品物流贸易基地。

　　(二)加快推进新型城镇化，增强现代化区域中心城市综合竞争力

　　着力构建大都市城镇体系。高起点编制和实施大都市全域规划。按照主城区近期200平方公里、200万人口，远期300-500平方公里、300万-500万人口规模，修编主城区城市总体规划；五年内主城区新增80平方公里城市新区，各县(市)新增20平方公里产业新区。把枣阳、河谷新城建成襄阳大都市的两个副中心城市，推进宜城、南漳与襄阳主城区一体化发展，把保康建成都市襄阳“后花园”和绿色襄阳示范区，把主城区周边具备条件的小城镇建成卫星城，建设一批特色中心镇和农村新型社区，对内形成中心城市、中等城市、特色小城镇、农村新型社区“四位一体”的大都市城镇体系；对外与周边城市联合互动，形成资源共享、功能互补、差异化发展的城市群。

　　着力完善城市功能布局。按照新城东进、旧城更新、一心四城、组群发展的战略，优化空间布局，完善功能分区。规划建设襄州新区、东津新区、樊西新区、襄南新区、庞公新区和隆中新区，实现200平方公里基础设施全覆盖。优化城市功能分区。鱼梁洲以汉水文化为主题，建成具有鲜明水生态特色的国际性旅游目的地。襄城重点发展以文化旅游业为形态的现代服务业，建成国内外知名的文化产业聚集地和旅游目的地。樊城重点发展以商务和高端商业为特征的现代服务业，建成辐射带动力强的区域性现代服务业中心。襄州重点发展现代生产性服务业，建成区域性现代物流中心和农产品生产、深加工产业基地。高新区、经开区重点发展战略性新兴产业和先进制造业，建成区域性高新技术产业中心和现代制造业中心。加快推进东津新城开发，五年内50平方公里起步区建设格局基本形成，把东津新城规划建设成为具有国际化水准、现代城市功能、承担区域中心城市辐射带动作用的新中心。

　　着力建设生态宜居城市。以创建全国文明城市、国家卫生城市、国家环保模范城市、国家生态园林城市和国家森林城市为抓手，加快建设生态宜居城市。实施城市生态建设工程，规划建设以鱼梁洲为“绿心”，襄南岘山森林公园、襄北连山湖公园、东津综合公园和襄城崔家营、老龙洲、长丰洲、贾洲、月亮湾、长寿岛等湿地公园为重点的生态片区，规划建设以汉江为纽带，唐白河、小清河、南渠、大李沟相贯通的五条生态廊道，引水入城，修复山体，构建城市生态功能体系，打造五百米见绿、一千米见园、两千米亲水的城市生态景观，促进城市与山水、江河、绿地和谐共生；高水平规划建设一批城市标志性工程、一批功能配套的城市综合体、一批具有鲜明特色的主题社区、主题公园、主题广场和便民利民设施。合理开发利用城市地下空间。坚持规划建设与管理并重，建立城市管理长效机制，推行数字化管理，持续开展门店招牌、户外广告、临街建筑立面、市政设施、交通秩序、沿街为市、拆墙透绿、建筑工地与渣土清运等八项集中整治，提高城市道路机械化清扫率、生活垃圾密闭收集率和垃圾无害化处理率，不断改善城市环境。

　　着力构建现代综合交通运输体系。建设与全国大通道直通直达、区域城际间方便快捷、区域内城乡交通一体、城区内交通通畅的综合交通运输网。重点建设十大交通工程。实施中心城区环线建设工程，规划建设沿江慢行环线，规划预留中心城区轨道环线，加快建设内环快速干线，构筑外环高速合围线。实施骨干路网建设工程，完善主城区干、支路网，形成“一心四城”互通对接的快速通道。实施中心城区交通畅通工程，完善组团式、网格化城市道路系统，高标准规划建设立交桥、人行天桥、地下通道、换乘枢纽和停车站场，开辟公交快速专线，优先发展公共交通，实现市区公交线路全覆盖，创建国家公交都市建设示范工程试点城市；加强交通综合管理，消减通行堵点，做好排堵保畅，力争达到国家“畅通工程”一等管理水平。实施高速公路主骨架建设工程，全面建成五条过境高速公路，实现县县通高速。实施公路干线提等升级工程，推进国、省道绕城改线等一批干线公路建设。实施国家公路运输枢纽工程，加快市、县、乡三级客货运站场体系建设。实施现代物流基地建设工程，建设襄阳综合物流信息中心，建设一批综合物流和专业物流园区。实施铁路运输功能提升工程，以北煤南运铁路专线建设为契机，布局煤炭集疏运系统，建设路口能源基地，争取规划建设郑渝高铁、西武高铁、襄武城际轨道交通，争取实现武汉至襄阳动车公交化运营。实施机场改扩建工程，把襄阳机场建设成为区域性门户机场。实施汉江航运体系建设工程，规划建设襄阳新港，加快建设新集、雅口航电枢纽，打造汉江中游航运中心和区域性水陆联运枢纽。

　　统筹推进城乡发展。加快推进城市生产要素和公共服务资源向农村延伸，促进城乡规划、产业发展、市场体系、基础设施、公共服务和社会管理一体化。抓好农村危旧房改造、环境整治、饮水安全、道路建设和电网改造升级，五年改造5万户农村危房，解决130万人的饮水不安全和90万个农户用电质量不高的问题，建成中心镇通达中心村、农村新型社区和产业化基地的交通网络。加快建设农村新型社区，按照“项目依托、产业支撑”的思路，建立政府引导、市场主体、农民参与的新机制，推进边远和弱小村向中心村集并，建成200个农村新型社区。加快建设中等城市和中心镇，增强承载和辐射带动功能；改革户籍管理制度，逐步解决农民工在城镇就业、居住、医疗、子女入学等方面遇到的困难，引导农村居民向城镇有序转移。突破性发展县域经济，力争有1个县(市)区冲刺全国百强，2个进入中部百强，3个进入全省10强，5个进入全省20强。

　　(三)加快发展文化事业和文化产业，增强文化软实力

　　大力弘扬时代精神，增强先进文化引导力。牢固树立“文化立市”理念，坚持以提升文化软实力增强城市竞争力。以创建全国文明城市为抓手，充分挖掘襄阳历史文化蕴含的艰苦创业、崇文重教、开放包容、重商亲商等主流价值，塑造新时期的襄阳人文精神、城市精神和发展文化，引导广大市民提升文明素质，优化人文环境，打造人文襄阳。

　　充分彰显城市特色，扩大历史文化影响力。坚持保护与利用、改造与复兴并重，推动襄阳丰富的历史文化遗产步入“以保护促利用、以利用促保护”的良性循环。充分发挥荆楚文化之源、三国文化之乡、汉水文化核心区的优势，加强保护和利用优秀历史文化遗产，充分挖掘和开发特色文化资源，延续城市历史根脉，擦亮襄阳文化名片，扩大城市影响力，彰显襄阳历史文化名城魅力。

　　加强公共文化建设，增强公共文化辐射力。以争创全国公共文化服务体系示范区为抓手，建立和完善覆盖城乡的公共文化服务体系。建成市区博物馆、图书馆、城市规划馆、群艺馆、大剧院，建成县(市)图书馆和文化馆，实现农村广播电视“户户通”。大力扶持文化精品创作生产，全国获奖文化精品数量继续保持全省前列。强化文化市场监管，确保文化事业健康有序发展。深化文化体制改革，加强文化人才队伍建设，激活文化生产力。

　　突破性发展文化旅游产业，提升区域文化竞争力。着力培育一批具有襄阳特色的文化产业集群，打造具有核心竞争力的知名文化企业。加快建设襄阳智谷文化产业园和汉城文化产业基地，规划建设三国文化城、米芾创意文化产业园和汽车文化创意产业园，加快建设襄阳古城、古隆中、岘山、鱼梁洲、鹿门山、保康九路寨、南漳古山寨、谷城大薤山、枣阳白水寺等文化旅游区，新建2个5A级景区、5个4A级景区、10家以上五星级酒店和一批四星级酒店，建设鄂西北旅游服务中心和旅游商品研发生产中心，把文化旅游业培育成新的支柱产业。建成中部地区旅游集散地、国内外有较大影响的旅游目的地和国家级文化产业示范区。

　　(四)加快建设“两型社会”，增强可持续发展能力

　　建设低碳城市。建立健全节能减排长效机制，扎实抓好低碳城市试点、国家可再生能源建筑应用示范城市、国家节能与新能源汽车示范推广试点城市建设，争取成为国家首批全国新能源示范城市之一，大力推进企业节能和建筑节能，在城市公交、市政、物流和出租车行业基本普及新能源汽车；倡导低碳生活，推广使用非化石能源和高效节能产品。实施主体功能区规划，形成层次分明的生态安全保障系统；加快构建“一城两带三网四区五廊”为主的城乡一体化生态系统，加强自然保护区建设，提高森林覆盖率和林木蓄积量，增加森林碳汇，建设鄂西北森林防火指挥、病虫害防治、石漠化治理、资源监测、林权交易和林业科研中心，建成国家森林城市。

　　建设资源节约型和环境友好型城市。实行最严格的耕地、林地和水资源保护制度，推进土地集约节约利用。加大地质灾害防治和区域恢复治理力度。科学开发利用磷、硅、钛等重要矿产资源，实现可持续发展。依法加强环境保护，严格执行建设项目环境影响评价制度和节能审查制度，杜绝新上高污染、高能耗项目；深入开展城乡环境综合整治，把汉江及其支流的水污染防治作为重中之重，切实抓好水体、大气、重金属和农业面源污染防治。建成国家环保模范城市。

　　构建绿色发展的产业结构。坚持以科技创新和技术进步推动节能减排，加快淘汰落后生产能力。大力发展可再生能源，着力构建安全、稳定、经济、清洁的现代能源产业体系。大力发展循环经济，抓好10个国家和省级循环经济试点、国家再生资源市场体系建设试点城市、谷城再生资源园区国家级“城市矿产”示范基地、襄阳磷化工循环经济产业园、高新区节能产业园和经开区再制造产业园建设，打造循环经济示范区。

　　(五)加快推进改革开放，增强跨越式发展的内生动力

　　加快构筑开放平台，着力建设中部地区开放高地。实施更加积极的开放战略，把对外开放与区域合作推上新高度。加快构建口岸大通关体系。建设一类航空口岸，设立水运口岸，完善铁路口岸和公路口岸功能，建设陆港口岸，优化开放平台。加快发展外向型经济。转变外贸发展方式，做大做强出口企业，优化出口结构，提高成套设备和高新技术产品出口比重。加大招商引资力度。更大范围地吸引和承接全球产业转移，与更多世界500强、中国500强、行业100强和中央企业开展战略合作，拓展利用内资和外资的广度和深度。抢抓日本、韩国和新加坡产业转移的重大机遇，建设日本、韩国、新加坡产业园，打造日、韩、新企业在中国重要的集聚地。抢抓海峡两岸经济合作框架协议实施的有利时机，建设两岸经济合作先行示范区，建成中部地区台商聚集地。

　　加大改革攻坚力度，着力突破体制机制障碍。大力推进金融改革创新。大力引进外资银行到襄阳设立区域总部或分支机构，争取商业银行总行在襄阳设立区域管理总部和后台服务中心。支持在襄金融机构向县(市)延伸服务机构。进一步推进农村信用社改革，支持其在各县(市)建设具有独立法人资格的农村商业银行，适时组建襄阳银行，进一步发展村镇银行和小额贷款公司。大力培育和发展地方非银行业金融机构。积极发展会计与审计服务、法律服务、资产评估、资质评级、投资咨询、理财服务等金融中介机构，支持发展中小企业信用担保机构。加快发展资本市场。大力支持企业上市，力争在襄阳上市公司达到30家以上，争取高新区成为代办股份转让系统试点；规范发展创业投资基金和创业投资公司，吸引内地股权投资企业投资和参股襄阳企业，吸引境内外股权投资机构在襄阳设立总部、分支机构和出资设立法人投资机构，支持具备条件的股权投资机构上市或发行企业债券，建立高新技术产业风险投资基金，各类股权投资基金发展到 50家以上；积极发展债券市场，鼓励支持符合条件的企业发行企业债券和融资债券，发行中小企业集合债券和集合票据。积极发展保险市场，大力引进保险机构总部或区域总部。探索发展要素市场。建设襄阳产权交易所、农特产品交易所等金融要素市场。加快发展票据市场，筹划构建票据业务信息系统。创新和完善政银企对接合作方式，优化金融生态环境，打造信用高地。积极推进行政管理体制改革和事业单位分类改革，深化医药卫生、食品药品监管、财税等体制改革。创新基本公共服务提供方式，扩大政府购买服务范围，实现提供主体和提供方式多样化。深化农村综合改革，创建全国农村体制机制创新试验区。

　　实施科教兴市和人才强市战略，着力建设创新型城市。加快建设区域性科技创新中心和科研成果产业化基地，以自主创新引领产业结构调整，推动科技、标准、产业同步发展。加快构建以高新区和经开区为龙头，县(市)开发区为支撑的区域创新体系，积极创建国家创新型特色园区；着力完善企业为主体、市场为导向、产学研相结合的技术创新体系，创建一批国家和省级实验室、检验检测中心、企业技术中心、工程技术研究中心，建立院士专家工作站、博士后工作站和博士后产业基地，构建技术联盟、产业联盟、标准联盟，研发和转化一批重大科技成果，培育一批创新型产业集群和创新型企业。坚持以用为本，建设区域性人才高地。实施高层次人才和基础人才培养工程，壮大各类人才队伍。深入实施“隆中人才支持计划”，设立“隆中人才发展基金”，面向海内外引进一大批创新创业团队、高端人才和紧缺人才，统筹建设各类人才队伍。创新人才工作体制机制，探索建立“人才特区”。

　　(六)加快推进改善民生为主的社会建设，增强民生保障能力

　　促进社会充分就业。广泛开展就业培训，保证新增劳动力全部接受岗前技能培训，全市在岗就业人员五年间普遍接受一次双技能培训。力争每年新增就业岗位10万个以上，促进城乡居民充分就业。坚持创业带动就业，以争创国家级创业型城市为契机，大力推动全民创业。完善各项创业扶持政策，建立创业扶持专项资金，扩大小额贷款担保资金规模。规划、建设和改造一批创业场所，建设一批创业基地。大力支持劳动密集型产业和小型微型企业发展，五年净增中小型企业和微型企业2万家以上，个体工商户5万个以上，拓宽就业渠道。完善政府购买服务机制，为就业困难群体提供更多的公益性岗位。逐步提高最低工资标准，千方百计增加居民财产性收入，确保城乡居民收入与经济增长同步。

　　健全统筹城乡的社会保障体系。提高城乡养老、医疗、失业、工伤和生育保险参保率，实现社会保险政策城乡全覆盖，城乡居民愿保尽保目标。推进机关事业单位养老保险制度改革。适时提高社会保障水平和统筹层次，努力实现人人享受基本社会保障。建立社会保险基金公共财政补助机制，稳步提高基本医疗保险报销比例、大病救助标准和离退休人员养老金待遇。完善以城乡低保为基础，专项资金相配套，临时救助为补充的社会救助体系，逐步实现城乡全覆盖。加大对困难群体的医疗、教育、就业、法律等专项救助力度。加强未成年人保护，切实保障妇女儿童合法权益，五年内通过政府投入、社会募捐等形式，新建800个以上农村留守儿童爱心服务站，组建万人“爱心妈妈”队伍，解决外出务工群众的后顾之忧。大力发展社会福利和慈善机构，完善以扶老、助残、救孤、济困为重点的社会福利体系。积极发展居家养老服务，建设一批老年公寓，壮大老龄服务事业和产业。做好移民安置后期扶持工作。

　　建立健全住房保障体系。扎实抓好保障性安居工程，五年内建成各类保障性住房7万套以上。其中新建廉租住房4000套，筹集公共租赁住房房源3万套，建设经济适用住房1.25万套，改造各类棚户区2.3万户以上，建设人才公寓1800套以上。扩大普通商品住房的有效供给。逐步解决城镇低收入群众、新就业职工和农民工住房困难。

　　深入推进扶贫开发。加大对贫困地区、革命老区扶持力度，抓紧编制和实施南河流域综合开发专项规划，扎实抓好秦巴山区全国集中连片扶贫开发重点县保康县的扶贫工作。完善扶贫开发机制，有效衔接扶贫开发与农村低保制度，每年帮助3万贫困人口脱贫致富，对南漳、保康、谷城三个山区县中缺乏基本生存条件的特困户分期分批实行扶贫搬迁，确保扶贫对象不愁吃、不愁穿，保障其义务教育、基本医疗和住房。

　　办人民满意的教育。实施学前教育公共服务体系覆盖工程，新建和改扩建幼儿园250所以上。实施义务教育学校标准化建设工程，改造、扩建、新建校舍150万平方米以上，基本实现县域义务教育均衡发展。调整优化普通高中布局结构，增加优质高中资源总量。完善特殊教育体系，新建、改扩建特殊教育学校8所。推进职业技术教育资源整合、集团化发展，组建职教联盟。促进在襄高校与国内外一流高校合作办学，支持襄樊学院创建一类综合性大学，支持襄樊职业技术学院创建国家示范(骨干)职业技术学院，加快发展襄阳汽车职业技术学院、襄樊学院理工学院和襄阳技师学院，力争在襄高校发展到7所以上，打造区域性教育中心。

　　大力发展卫生事业。健全覆盖城乡的医疗卫生服务体系。规划建设区域性医疗服务中心、公共卫生服务中心、妇女儿童保健中心、中医药医疗中心、残疾人康复中心、疾病防治中心、鄂西北工伤康复中心等重大项目。健全疾病预防控制、健康教育、应急救治等公共卫生服务网络，提高重大疾病和突发公共卫生事件预防处置能力。加强惠民医院建设，逐步改造95个乡镇卫生院、110多个城镇社区卫生服务中心(站)和2300多个村卫生室，确保政府举办的基层卫生机构全部达标。实施全民健康行动计划，提高全民健康水平。完善全民健身服务体系，完成全民健身二期工程，广泛开展全民健身活动，增强全民体质。健全人口和计划生育公共服务体系和奖励扶助制度，稳定低生育水平，促进优生优育，提高出生人口素质，遏制出生人口性别比偏高趋势。

　　加强和创新社会管理。着力完善党委领导、政府负责、社会协同、公众参与的社会管理格局。全面建立城镇社区和农村新型社区公共服务站，健全网格化、信息化、多元化、专业化的服务管理体系，培育和发展各类社会组织，提高城乡社区自治和服务功能。健全完善社会矛盾大调解体系，建立干部下基层、接访常态化工作机制和矛盾纠纷多元调处机制，畅通群众诉求表达、利益协调、权益保障渠道。实行重大决策事项社会稳定风险评估，从源头上预防和化解社会矛盾。创新流动人口和特殊人群服务管理，努力增加和谐因素。健全完善社会治安防控体系，严厉打击各类犯罪，纵深推进打黑除恶专项斗争，增强公共安全和社会治安保障能力，努力建设最具安全感城市。夯实安全生产基层基础，严格落实安全生产责任制，防控重特大事故发生，创建本质安全型城市。健全产品质量和食品药品监管机制，让群众用得安心，吃得放心。完善突发公共事件应急管理体系，提高预防控制和应急保障能力。加强国防后备力量建设和人防工作，巩固和发展军政军民团结，继续创建“全国双拥模范城”。做好新形势下的民族和宗教工作，促进民族团结和谐。

　　三、关于2012年的主要工作

　　今年全市经济社会发展的预期目标是：生产总值增长15%，规模以上工业增加值增长23%，全社会固定资产投资增长40%，社会消费品零售总额增长20%，实际外商直接投资和出口额分别增长20%，地方一般预算收入增长22%，城镇居民人均可支配收入和农民人均纯收入分别增长11.2%和11.4%；城镇登记失业率控制在4.5%以内。全面完成省政府下达的物价调控、节能减排、计划生育和安全生产等约束性指标。

　　围绕实现上述目标，我们将重点做好七个方面的工作：

　　(一)着力做大做实做强投资，加快培植发展后劲

　　坚定不移地把招商引资和项目建设，作为培植发展后劲的关键举措，确保全年完成全社会固定资产投资1500亿元。强力推进招商引资。紧紧围绕“一个龙头、六大支柱”产业，着力在引进行业龙头企业和企业总部上实现突破，促进与中央企业签约项目落地。全年新注册投资过100亿元的工业项目2-3个、过50亿元的5-6个、过10亿元的50个。抢抓中央实施积极财政政策的重大机遇，加大中央投资项目和资金的争取力度。强力推进重点项目建设。确保投资5亿元以上在建项目达到100个以上，其中投资过100亿元的项目5个以上、50亿元以上的10个、10亿元以上的50个，全年开工投资过100亿元或产出规模过100亿元的项目12个，突出抓好东风股份高端商务车、特种车和汽车零部件、东风德纳车桥、北汽福田专用车、国通青扬新能源纯电动客车和动力电池、惠浦创新电子液晶显示器、康明斯发动机、珠江数控、江苏爱康太阳能光伏发电、兴发集团和龙蟒集团磷化工等一批重大产业项目建设，全力促进汉江流域综合开发、襄阳新港、新集航电枢纽、襄北铁路编组站、谷竹和老谷高速公路、保宜和麻竹高速公路襄阳段等一批重大基础设施项目建设。

　　(二)着力做大做实做强产业，强化跨越式发展支撑

　　深入实施工业跨越式发展行动计划。以改造提升传统产业，突破性发展高新技术和战略性新兴产业为重点，积极促进工业化与信息化融合，强力推进工业项目建设，确保工业投资突破900亿元，全力促进“一个龙头、六大支柱”产业做大做强。全年规模以上工业产值突破4000亿元，其中汽车及零部件和农产品加工业产值分别突破1500亿元和1200亿元，装备制造、电子信息、医药化工产业产值分别突破200亿元，新能源新材料产业产值突破100亿元。加快推进骨干企业做大做强，新增3家产值过50亿元、5家过10亿元、100家过亿元的企业。全面落实扶持政策，促进中小企业和微型企业成长壮大，全年新增工业企业1000家、规模以上企业100家。加快建设产业集聚区，实施高新区40平方公里、经开区50平方公里和县(市)开发区20平方公里产业新区基础设施覆盖工程，建设襄阳科技城，着力构建功能完善的产业集聚平台。启动建设国家汽车零部件检测重点实验室、动力电池产品质量监督检验中心和区域性食品药品检验检测中心，优化产业配套环境。突破性发展现代服务业。深入实施限上商贸企业成长工程，支持骨干商贸企业做大做强；改造提升传统商业网点，启动改造一批主城区菜市场，建设一批社区平价菜店，打造社区“8分钟生活圈”；启动12个中心镇流通现代化建设试点，完善农村流通网络。积极发展高端服务业，启动建设中国汽车服务外包基地和区域性金融服务外包基地，启动建设华强中央文化商务区等10个以上大型城市综合体，加快建设中国移动湖北公司襄阳创新基地“四个中心”项目。大力发展现代物流业，开工建设襄北和樊西综合物流园、襄阳农产品物流中心、烟草物流园，启动建设北煤南运煤炭储运配送中心、郜营铁路货运中心和余家湖水陆联运物流园区，建成现代物流信息中心和汽车产业物流园区。加快发展现代农业。突破性发展农产品加工业，加快建设正大集团百万头生猪养殖加工、际华3542公司家纺生产线等8个总投资110亿元的重大项目，推进白酒和米、面、油加工行业整合，重点支持奥星粮油、襄大农牧、襄阳正大、湖北梅园等骨干企业做大做强，促进农产品加工业提档升级。加快发展规模化农业，扎实推进粮食增产工程，力争跨入全国百亿斤粮食大市行列，抓好“菜篮子”工程和国家油菜产业基地建设，建成一批省级畜牧水产标准化养殖示范场，扩张烟叶、茶叶、水果等特色产业，扩大生物质能源林、高效经济林、速生丰产林等林业板块基地。着力提升农业综合生产能力，突出抓好引丹灌区等大中型灌区续建配套和节水改造，以深入开展“三万”活动为抓手，大力推进“小农水”建设；做好“引丹入襄”工程前期准备工作。健全基层农业服务体系，加大实用技术推广和优质高产创建力度，加快实施全国农业机械化示范项目，创建鄂西北农业科技创新服务中心，促进农业科技进步。规划建设襄州万亩现代种植业示范区、农产品加工园区和物流园区，打造现代农业示范区。

　　(三)着力推动文化改革发展，加快建设文化强市

　　大力推进公共文化设施建设，启动建设博物馆、大剧院和城市规划馆，完善公共文化服务体系，增强公共文化服务功能。深入开展文化惠民活动，着力培育“十里汉江文化长廊”和“欢乐襄阳”等群众文化活动品牌。加强文化人才队伍建设，鼓励创作一批具有浓郁襄阳文化特色的文艺精品。突破性发展文化旅游产业，加快建设襄阳智谷文化产业园，启动建设文化科技产业园和汉城文化产业基地；加快推进重点文化旅游区建设，加快修复襄阳古城、习家池等历史文化遗迹，积极做好襄阳古城申报世界文化遗产工作，启动建设鱼梁洲汉水文化旅游综合试验区；充分挖掘“中国红嘴相思鸟之乡”的品牌价值，大力发展生态旅游，打造“生态浪漫之都”；积极发展体育休闲旅游产业，争创国家级航空运动中心和水上运动中心；新建2家五星级和2家四星级酒店。强化文化旅游营销，确保旅游收入突破150亿元，文化产业增加值占生产总值的比重高于全省平均水平。加快推进文化体制改革，增强文化事业和文化产业发展活力。

　　(四)着力提升城市功能，推进城乡统筹发展

　　科学编制城市规划。完成200平方公里主城区城市总体规划修编和申报工作；完成襄阳大都市城镇体系规划、襄阳城市空间发展战略规划、东津新城概念性规划及核心区城市设计，做好襄州新城规划和滨江风光带、庞公新区的城市设计。强力推进城市重大项目建设。启动建设东津新区、庞公新区、隆中新区、襄南新区和襄州中央商务区，重点推进东津新城50平方公里起步区开发；加快建设城市交通设施，重点抓好内环南线、东线等70个交通路网项目建设，推进国道绕城工程、城市桥梁工程、断头路打通工程、长虹路综合整治工程和主次干道刷黑工程，确保汉江卧龙大桥和唐白河大桥建成通车，缓解城市交通拥堵；加快构建综合交通运输网，建成襄阳火车东站，增加襄阳至武汉动车班次，增开襄阳至国内大城市的航班、航线，加快改造国、省干线公路、绕城公路和县乡等级公路，启动建设襄阳汽车客运东站和南站。着力改善城市人居环境。大力推进旧城改造和城中村改造，实施康居工程，改善居住环境。以创建国家森林城市为抓手，统筹推进城乡生态建设，扎实抓好城乡造林绿化和天然林保护二期工程；加快实施主城区岘山森林公园等11个生态景观项目，启动建设汉江、南渠和小清河生态廊道，推进汉江一桥至鱼梁洲段整体开发，打造“亲山近水”的城市生态景观；抓好主城区29个防汛排涝项目建设，配套完善城市排水管网。以“五城同创”为抓手，深化“城管革命”，强力推进城市环境“八项集中整治”，着力解决垃圾围城和市民如厕难的问题，深入持久地打好城市查处违法建设攻坚战，确保违法建设“零新增”，促进城市环境显著改观。大力推进节能减排。认真落实地方政府和市直部门保护汉江责任制，抓好主城区8个环境保护项目建设，完成张湾和襄城截污干管改造，建成余家湖污水处理厂、隆中截污干管和泵站，加快建设县(市)垃圾处理厂和污水处理厂，严格执行排污许可制度，确保汉江干流襄阳段水质稳定在现有水平，促进支流水质进一步改善；加快淘汰落后产能，抓好百家重点耗能企业节能工程，新增可再生能源建筑应用面积85万平方米，推广使用各类新能源汽车200台以上，降低城市污染指数。着力统筹城乡发展。完成县(市)区全域规划、县级城市新区规划、中心镇规划和村镇体系规划；加快建设县级城市和中心镇基础设施，增强承载和辐射能力；以建设县(市)产业新区为抓手，推进县域工业向开发区集中，打造城乡一体化产业平台；以建设农村新型社区为抓手，综合利用国家农业综合开发、土地整理、水利建设、环境整治、建材下乡、危房改造、电网改造、交通建设、现代流通网络建设等政策，集中力量推进农村基础设施建设，加快构建综合性公共服务平台；全市集中力量办好襄州区统筹城乡发展综合试点，各县(市)区以城乡结合部和中心镇为重点办好1-2个试点。

　　(五)着力推进改革开放，创新跨越发展的体制机制

　　加快对外开放步伐。着力构筑开放平台，启动襄阳机场改扩建，申报一类航空口岸，大力发展口岸物流，优化对外开放环境；着力打造国家级家纺出口基地和省级农产品出口基地，创建国家级汽车零部件出口基地，努力扩大出口规模。大力推进金融创新。积极推进优质企业与创投公司、风投公司、股权投资基金合作，与资本市场对接，努力扩大直接融资规模，在债券融资和融资租赁上取得突破，争取证监会受理5家企业上市申请，促进10家企业进入上市辅导期，争取高新区代办股份转让系统挂牌运行。深化投融资体制改革。组建襄阳城投控股集团，争取与国开行基金公司合作建立投资基金。积极推进财税改革。创新税收征管机制，加大社会综合治税力度，促进财税收入持续增长；整合中央和省市财政专项资金，加大对“三农”、保障性住房、社会事业等民生领域的投入；完善财政专项资金的导向机制，引导更多的社会资本投入实体经济、社会事业和基础设施建设。深化重点领域和关键环节改革。完善户籍制度改革配套政策，形成科学有序的转户常态机制；加快社会事业和公共服务领域改革，积极推进事业单位分类改革；深化农村综合改革，继续抓好集体林权制度配套改革，推进国有林场改革，实施农业水价、水管体制和小农水产权制度改革，创新土地流转机制。大力发展农民专业合作社，完善农业社会化服务体系。加快创建国家创新型城市。支持高新区抓好国家级创新型特色园区建设，创建国家科技与金融结合试点园区；着力提升企业创新能力，建成5家省级工程技术研发中心、企业技术中心和校企共建研发中心，新增5家博士后工作站和5个博士后产业基地，培育省级创新型企业25家，实施产学研合作项目70个以上，申请专利4000件以上，申报国家驰名商标5件以上。创新人才工作机制，培育提升人力资本。大力实施“隆中人才支持计划”，面向海内外引进一流的产业创新团队10个以上，引进硕士、博士等高层次人才400名以上；大力发展高等教育和职业技术教育，力争在推进在汉高校、国外高校与在襄高校合作上实现突破，支持襄樊学院设立硕士学位点，促进襄樊职业技术学院通过国家示范(骨干)院校评估验收，建成襄阳汽车职业技术学院和襄樊学院理工学院新校区，加快建设襄阳技师学院和公共实训基地，规划建设职业教育产业园区，组建职业教育集团，优化人才培养平台。

　　(六)着力创新社会管理，促进社会和谐稳定

　　创新社会管理体制机制。健全完善群众利益协调、诉求表达、权益保障和社会稳定风险评估机制。完善县乡村三级联动矛盾调解网络，健全社会矛盾大调解体系，提高社会矛盾化解能力和化解效率，妥善解决群众合法合理诉求，坚决纠正损害群众利益的行为。以乡镇综治维稳信访联动中心为基础，成立乡镇社会管理服务中心，强化整体联动功能。加强社区公共服务站建设和网格化管理，努力实现信息全收集、服务全到位、管理全覆盖、事态全掌控。推进社会管理综合信息平台向县(市)和乡镇延伸，提高社会管理信息化水平。建立流动人口特别是对农民工的服务管理机制。完善应急管理体系，提高应对公共危机和突发事件的能力；健全安全生产和产品质量责任体系，提高安全生产和产品质量监管能力；深化食品药品管理体制改革，完善分段管理、无缝衔接的管理机制，建立食品药品质量追溯制度，严厉打击食品药品领域的违法犯罪行为，实施“早餐示范工程”，争创全国“早餐示范工程”试点城市。完善防灾救灾体系，建设区域性防灾救灾指挥和物资储备中心。加强社会治安综合治理，严厉打击各类违法犯罪，持续开展打黑除恶，扎实抓好新一轮“全国综治优秀市”和“全省平安市”创建工作，进一步增强人民群众安全感。

　　(七)着力保障和改善民生，提高人民群众幸福指数

　　千方百计扩大就业。加强就业指导、信息服务和技能培训，完善就业援助机制，重点做好高校毕业生、农民工、困难群体和退役军人的就业工作。全面落实创业扶持政策，深入推进全民创业，促进创业带动就业。确保城镇新增就业10万人，下岗失业和困难人员就业3.9万人，转移农村劳动力就业6万人。进一步完善社会保险、社会救助和住房保障体系。推进社会保险扩面征缴，全面实施医疗、工伤和生育保险市级统筹，启动实施社会保险“一卡通”惠民工程；规范完善最低生活保障制度，健全社会救助标准与物价上涨联动机制，加大对困难群众重特大疾病医疗救助力度，全面推行医疗救助即时结算；大力发展慈善和社会福利事业，加大扶老、救孤、助残力度，加快建设襄阳老年公寓，启动建设市社会福利中心和军休服务中心，建成200个农村留守儿童爱心服务站；扎实推进保障性安居工程，向城镇人均住房建筑面积13平方米以下的低收入家庭发放租金补贴；坚决落实中央关于房地产市场调控的各项措施，促进房地产市场稳定健康发展。加快发展社会事业。坚持优先发展教育，全面实施学前教育三年行动计划和县市区义务教育均衡发展三年行动计划，加快推进“农村中小学校舍安全工程”；资助贫困学生13万人以上。深化医药卫生体制改革，巩固和完善基本药物制度，试行县域医疗机构集团化，完善乡、村卫生机构一体化管理；推进基本公共卫生服务均等化，提高传染病防治和紧急医疗救援水平，积极发展妇幼保健、职业病防治、无偿献血和中医药事业，扎实推进全民健康行动。加强人口与计划生育工作，狠抓出生人口性别比专项治理，稳定低生育水平。

　　在做好以上各项工作的同时，市政府将集中力量办好关系民生的十件实事。

　　1.全面建立城乡居民社会养老保险制度，实现社会养老保险制度全覆盖。

　　2.开工建设各类保障性住房1.27万套，改造城市棚户区1万户，缓解城镇中低收入家庭住房困难。

　　3.开工建设博物馆、大剧院(含音乐厅)、城市规划馆和全民健身活动中心(二期工程)；新建城市社区流动图书站30个。

　　4.实施城乡义务教育学校标准化建设工程，达标学校达到150所；大力发展学前教育，新建改建城乡幼儿园50所，确保每个乡镇有一所公办幼儿园。

　　5.创建10个示范乡镇卫生院(社区卫生服务中心)和100个示范村卫生室。

　　6.疏浚治理全市1.38万口堰塘；解决20万人饮水不安全问题；改造农村危房10000户以上，搬迁贫困户800户以上，再解决3万贫困人口的温饱问题。

　　7.新建改建城市标准化社区服务中心40个，实现城市社区管理服务网格化；完成国有破产改制企业水电改造5000户。

　　8.贯通内环西线；开工建设长征东路清河桥、富康路清河桥；打通春园北路、唐家巷南路、女贞路、三元北路等4条断头路；完成三元路等10条道路改造；新建三处过街通道；新开城市公交线路4条，日增加城市公交班次200次，延伸公交路线80公里，新建改建20处港湾式站台；新建城区停车场5个。缓解城区交通堵塞压力。

　　9.实施40条背街小巷硬化、亮化、绿化工程，改造人行道15万平方米，建设6座生活垃圾转运站，新建4个2000平方米以上的标准化菜市场。

　　10.启动建设市残疾人康复技术资源中心；新建200所市级留守儿童爱心服务站；继续实施0-6岁残疾儿童康复工程。

　　四、关于政府自身建设

　　建设新襄阳，实现新跨越，是历史赋予我们的新使命，是全市人民的根本利益所在。我们将勇担历史重任，坚持执政为民，以奋发有为的精神状态和务实高效的工作作风，切实加强政府自身建设，努力成为让人民满意和放心的政府。

　　(一)深入解放思想，建设创新型政府。坚持把改革创新贯穿于工作全过程。创新政府领导经济工作的方式，立足襄阳实际，遵循发展规律，敢于先行先试，自觉运用市场经济的手段和方法推动经济工作，以改革创新的举措破解各种瓶颈制约，在各个领域探索具有襄阳特色的发展模式。创新政府工作决策方式，建立重大决策协商和征求意见制度，健全重大事项专家咨询、社会公示、公示听证制度，提高决策开放性，促进决策科学化。扎实推进学习型机关建设，加大公务员培训力度，不断提高谋发展、解难题、闯新路的认知水平，不断增强创新举措推进发展的能力。

　　(二)优化行政服务，建设效能型政府。加快转变政府职能，优化机构设置，逐步实现扁平化管理，加快建立与国际接轨、与发达地区无差异、与市场经济体制要求相适应的政府管理体制和服务方式。推进“阳光新政”工程化，加快行政服务标准化和信息化步伐，在政府机关率先运用云服务，建设“公共服务云”。深化政务公开，规范行政行为；深化行政审批制度改革，精简审批事项，优化审批流程，大力推进集中审批和并联式审批，提高审批效率。加快建立科学的行政责任体系，建立和完善层级清晰、具体量化的能力席位标准。加强目标管理，完善激励机制，强化效能监察，集中治理庸懒散软，切实提高执行力，做到有令必行、有禁必止，雷厉风行、真抓实干。切实加强政风建设，全心全意为人民群众服好务，坚持把群众的呼声、意愿和诉求作为施政理政的“定盘星”，决策尊重民意，绩效由民评判。从体制机制入手，着力解决征地拆迁、劳动争议、环境污染等群众反映突出的问题，办好顺民意、解民忧、惠民生的实事。

　　(三)坚持依法行政，建设法治政府。牢固树立依法治市的理念，深入实施“六五”普法规划，提高全社会法律意识。严格依法行政，规范行政处罚裁量权，将权力置于人民监督下阳光运行。深化行政综合执法改革，强化城乡建设、社会治安、安全生产、产品质量、食品药品安全等领域行政执法，切实做到严格、规范、公正、文明。严格执行执法责任制和执法违法责任追究制。加强行政复议和行政调解，扩大政府信息公开，推行网络政务，加强网络监督。自觉接受市人大、市政协监督和舆论监督、社会监督，确保行政权力依法、公正、有效运行。认真办理人大代表议案、建议和政协委员建议案、提案。积极听取民主党派、工商联、无党派人士和人民团体的意见建议，提高政府依法行政的能力和水平。

　　(四)加强廉政建设，建设廉洁政府。始终牢记“两个务必”，恪守廉洁从政若干规定。实施源头治理，以制度规范和阳光运作，堵住公共资源交易、公共资金管理、工程招投标等腐败易发多发领域的漏洞。严格“三公”管理，建设节约型机关。坚持从严治政，严肃查办领导干部滥用职权、贪污贿赂、腐化堕落、失职渎职案件，严肃查办商业贿赂和严重侵害群众利益案件，严肃查办群体性事件和重大责任事故背后的腐败案件，严肃查办为黑恶势力充当“保护伞”的案件，绝不让违法违纪行为干扰改革开放大局，绝不容腐败分子玷污政府形象。

　　各位代表！襄阳正在崛起，前景催人奋进。让我们在市委的坚强领导下，倍加珍惜不可多得的黄金机遇期，倍加珍惜来之不易的发展好势头，倍加珍惜心齐气顺、风正劲足的发展大气场，团结带领全市人民，同心同德，扎实苦干，为开创襄阳跨越发展新局面、谱写人民美好生活新篇章而努力奋斗！
